# The arc of Mass Spectrometry Exchange Formats is long, but it bends toward HDF5

**DOI:** 10.1002/mas.21522

**Published:** 2016-10-14

**Authors:** Manor Askenazi, Hisham Ben Hamidane, Johannes Graumann

**Affiliations:** ^1^ Biomedical Hosting LLC Arlington MA; ^2^ Research Division Weill Cornell Medicine in Qatar Doha State of Qatar

**Keywords:** mass spectrometry, data exchange formats, HDF5

## Abstract

The evolution of data exchange in Mass Spectrometry spans decades and has ranged from human‐readable text files representing individual scans or collections thereof (McDonald et al., 2004) through the official standard XML‐based (Harold, Means, & Udemadu, 2005) data interchange standard (Deutsch, 2012), to increasingly compressed (Teleman et al., 2014) variants of this standard sometimes requiring purely binary adjunct files (Römpp et al., 2011). While the desire to maintain even partial human readability is understandable, the inherent mismatch between XML's textual and irregular format relative to the numeric and highly regular nature of actual spectral data, along with the explosive growth in dataset scales and the resulting need for efficient (binary and indexed) access has led to a phenomenon referred to as “technical drift” (Davis, 2013). While the drift is being continuously corrected using adjunct formats, compression schemes, and programs (Röst et al., 2015), we propose that the future of Mass Spectrometry Exchange Formats lies in the continued reliance and development of the PSI‐MS (Mayer et al., 2014) controlled vocabulary, along with an expedited shift to an alternative, thriving and well‐supported ecosystem for scientific data‐exchange, storage, and access in binary form, namely that of HDF5 (Koranne, 2011). Indeed, pioneering efforts to leverage this universal, binary, and hierarchical data‐format have already been published (Wilhelm et al., 2012; Rübel et al., 2013) though they have under‐utilized self‐description, a key property shared by HDF5 and XML. We demonstrate that a straightforward usage of plain (“vanilla”) HDF5 yields immediate returns including, but not limited to, highly efficient data access, platform independent data viewers, a variety of libraries (Collette, 2014) for data retrieval and manipulation in many programming languages and remote data access through comprehensive RESTful data‐servers. © 2016 The Authors. *Mass Spectrometry Reviews* published by Wiley Periodicals, Inc. Mass Spec Rev 36:668–673, 2017

## I. INTRODUCTION

As a general rule, mass spectrometers produce output stored in manufacturer‐specific proprietary file formats. A combination of the need to efficiently store ever larger data sets, regulatory requirements for tamper‐proofing in the context of medical trials and the manufacturer's tendency to lock data produced on their platform into an exclusive analytical ecosystem lead to a largely binary on‐disk representation. Beyond the inherently reduced accessibility of proprietary binary files (which cannot be accessed without prior knowledge of their structure and/or dedicated tools) the formats and tools used are often burdened with requirements for backward compatibility bridging decades, as well as software dependencies precluding the use of vendor‐provided tools on UNIX‐like systems central to many high‐performance data analytic pipelines.

In response to this situation, the proteomics community set about to define a controlled vocabulary for mass spectrometry data as well as a standard format for data interchange. It settled on the PSI‐MS for the controlled vocabulary: expressed as a 17,000 Line OBO v1.2 file (Open Biomedical Ontologies [Smith et al., 2007]) this extremely detailed and increasingly comprehensive formal standard defines and inter‐relates most key concepts in the field of mass‐spectrometry and mass‐spectrometry‐based proteomics (e.g., MS:1000628 is the formal accession for a “basepeak chromatogram” which is a kind of MS:1000810, i.e., “mass chromatogram”). Any storage and interchange format aiming to become a standard must provide a mapping from its various data‐fields to these concept‐codes. The official data interchange format that was selected by the community, namely mzML (Deutsch, 2008), does indeed provide this mapping, and does so in every instance (i.e., in every individual mzML file). This is because the PSI community desired the standard to be self‐describing, as well as universally supported and human readable. Collectively, these requirements led to the choice of XML as the underlying representation technology for the mzML format.

## II. SCALING BEYOND HUMAN READABILITY

Following the adoption of mzML by the mass spectrometry community, a gulf of ever increasing size has emerged between the performance characteristics of XML and the requirements of sharing and efficiently accessing data sets: software systems built around the XML‐based format (as well as the related mzXML [Pedrioli et al., 2004] format) suffer from a mismatch between the data being stored and the storage format being used, with a resulting penalty in terms of file size and performance characteristics. In order to maintain the positive gains of the adopted format (in the form of desirable features such as universality and human readability), the developers of mzML compliant tools have had to engineer increasingly complex adjunct software in order to keep the resulting systems performant. Examples of this include the storage of image data in adjunct files external to the main mzML file in imzML (Römpp et al., 2011), the generation of external binary files (“.cachedMzML” files) by OpenMS to support efficient access to SWATH data stored in mzML (Röst et al., 2015), and the incorporation of the MS‐Numpress (Teleman et al., 2014) compression schemes into ProteoWizard (Chambers et al., 2012) to mitigate the up to 18‐fold inflation in mzML file size as compared to the original vendor format (Teleman et al., 2014). These software engineering fixes are all symptomatic of the technical drift arising from the choice of a text‐based format for the storage of increasingly large volumes of mass spectrometry data.

## III. HDF5 IS A NATURAL CHOICE FOR LARGE SCIENTIFIC DATASETS

The Hierarchical Data Format version 5 (HDF5) is an open source, binary file format for storing large and complex datasets, and is particularly suited for scientific and technical applications. The format supports extremely efficient access to and manipulation of complex and hierarchical data while maintaining universality both in terms of supported architectures, operating systems and programming languages. The format effectively offers facilities for an unlimited variety of data types, thanks in part to its self‐describing nature. This in turn implies that computer systems accessing a file can adjust and optimize access patterns to the specific (potentially unique) data type encountered. Work on the format began at the NCSA in 1987 and has since been funded by the NSF, NASA, and the DOE. It has been heavily adopted by many US national labs including Lawrence Livermore, Los Alamos, and Sandia National Laboratories. Over the years, a thriving software ecosystem has emerged around the format including data viewers, programming language support, and parallel file processing to support massively parallel high performance computing environments.

## IV. OpenMSI AND mz5—HDF5 PIONEERS IN MASS SPECTROMETRY

The shortcomings of XML were perhaps most keenly felt in the domain of imaging mass spectrometry where even the first proposed standard for this sub‐domain of mass spectrometry (imzML [Römpp et al., 2011]) abandoned the notion of a pure XML format. The raw data produced by these experiments is, in fact, never stored in XML but rather in a separate imaging binary data file (.ibd file). The overall format is still considered a variant of mzML since the metadata for the experiment is stored in “classic” mzML with empty binaryDataArray tags referring to the external “.ibd” file. In response to this situation, and in in order to better support parallel data access, the OpenMSI (Rübel et al., 2013) format was defined, which is based entirely on a single HDF5 file. The format leverages HDF5's built‐in gzip support and achieves excellent storage efficiency as well as support for both parallel read and write operations. The metadata and provenance information are also stored in the same unique HDF5 file without disrupting the data format thanks to the extensibility and self‐describing nature of the format.

**Figure 1:**
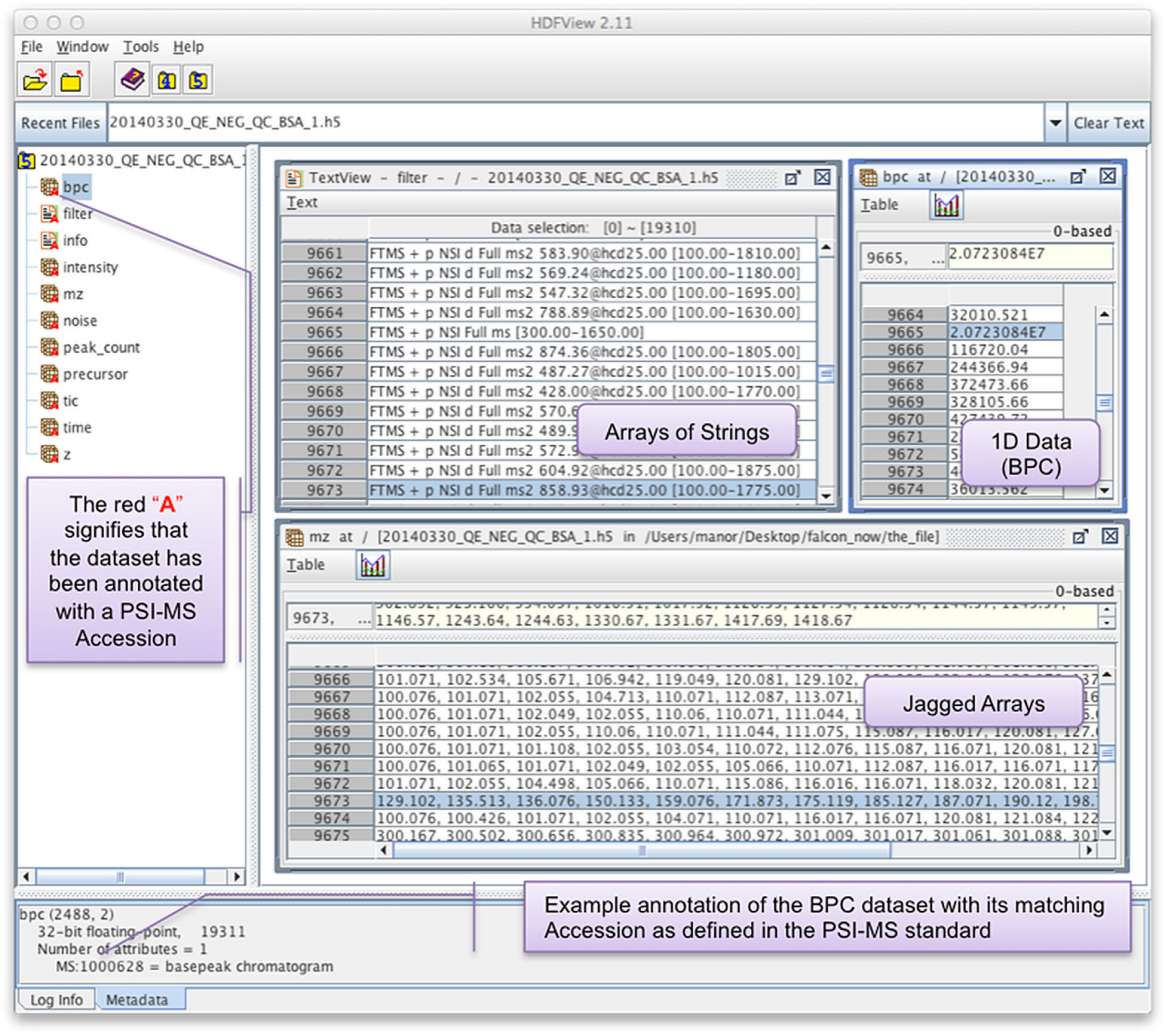
HDFView enables cross‐platform access to a Shaduf generated HDF5 file.

Another instance of HDF5 being used in mass‐spectrometry was the incorporation of support for mz5 (Wilhelm et al., 2012) in ProteoWizard as an alternative storage model for mzML. The idea behind this format is that software which accesses raw mass spectrometry data through ProteoWizard can be made to use HDF5 behind the scenes without any change to the high‐level application programming interfaces (APIs) used by the programmer. The benefit of this approach is a significant increase in storage and data access performance with no disruption to the sizable existing software base. Unfortunately, the proposed mz5 encoding of the raw data does not fully leverage the self‐describing nature of HDF5. In pursuit of optimal performance the authors of the format adopted, for example, a delta mass representation, which, while yielding smaller data files, does reduce their accessibility by standard software/viewers (which rely solely on the data‐typing explicitly declared in the file format). As a result, it is not possible to, for example, browse an mz5 data file using the standard HDFView data viewer (in contrast to a straightforward representation which is trivially accessible as seen in Fig. 1).

## V. ANNOTATING HDF WITH THE PSI‐MS CONTROLLED VOCABULARY (CV)

A positive aspect of mz5's 1‐to‐1 compatibility with mzML is the reuse of PSI's comprehensively curated controlled vocabulary for mass spectrometry (henceforth, PSI‐MS CV). Every data element in mz5 is annotated with an PSI‐MS CV accession, which gives it a definitive meaning, that can be referenced by humans and software alike. We hypothesize that a straightforward encoding of mass spectrometry data in HDF5 coupled with annotations using the PSI‐MS CV can serve as a natural evolution away from XML as the underlying storage format in mass spectrometry. We have initiated a research program to assess this strategy by experimenting with a range of format designs all having in common a few key characteristics: (i) datasets must have self‐explanatory names and be encoded in efficient yet standard representations of data (binary vectors of doubles wherever possible with names such as “mz,” “intensity,” or “noise”); (ii) we leverage yet another extremely useful feature of HDF5, namely the ability to associate attributes with datasets, in order to tag each dataset with the ID (“PSI‐MS_ID”) and name (“PSI‐MS_NAME”) of the CV term which best describes it; and (iii) in cases where PSI has not issued an appropriate controlled term, we fall back on a more general parent term which will apply while being necessarily over‐generalized. For example, when storing noise information per centroid as provided by Thermo Fisher Scientific (Waltham, MA), we lack a term for noise array (not to be confused with the signal‐to‐noise array which does have an official accession, MS:1000517). In this situation we fall back on “binary data array” (MS:1000513).

The simplicity of the approach is most evident upon opening an HDF5 dataset in, for example, the standard and cross‐platform tool HDFView (Fig. 1). It is immediately obvious what datasets are being provided and what their types are—this is because we store all the data in ragged‐arrays that store each element type (e.g., *m/z*, intensity, charge state [*z*], noise etc.) across all the scans acquired in the dataset. For complex acquisitions (e.g., when attempting to identify small molecules *via* hierarchical product ion spectra scan types, aka scan trees) HDF5 may be used through another one of its features, namely the ability to create a hierarchy of groups—these can naturally represent the tree‐like nature of many product ion spectrum (MS^n^) acquisitions. While in the regular format annotations with PSI‐MS CV accessions may be made for all scans in a single non‐redundant location, the hierarchical mode allows for independent annotation of every individual element, as in the mzML data model. Note that Shaduf, our HDF5 generating tool (described below) is able to generate both styles of HDF5 (the default being ragged mode, and the alternative being hierarchical mode).

## VI. HDF5 SUPPORTS THE EVOLVING FUNCTION OF DATA INTERCHANGE FORMATS

Clearly, we are not yet in a position to propose a complete HDF5‐based standard. We suggest that a period of trial and error should be considered, somewhat analogously to the current strategy of the PRIDE repository (Martens et al., 2005)–which today accepts a very broad range of data files where it initially mandated rather strictly which exact data formats should be uploaded. This tolerance to variation will serve the community well as, for example, we discover that certain data vectors are worth storing routinely (e.g., “signal to noise array,” MS:100517 has assumed much greater importance in quantitative Orbitrap experiments following the publication of observations by Makarov and Denisov (2009). Furthermore, as leading journals in the field restore the mandate to deposit all raw data in public repositories (Burlingame et al., 2015) and as repositories enable authors to upload raw vendor data, the function of the interchange data format may shift: authors will comply by uploading their raw vendor data files and use the standard interchange format to communicate a more useful subset of the underlying data (e.g., the authors may upload the full profile data in its original RAW file, while keeping only the vendor‐defined centroids in the HDF5 file).

## VII. PROOF OF CONCEPT IMPLEMENTATION: SHADUF

Shaduf is a python script that can export Thermo Fisher Scientific Raw files into a ragged‐array based HDF5 format for mass spectrometry data, without the need for ProteoWizard. The script requires Python including the NumPy and h5py extensions, as well as a copy of the MSFileReader (https://thermo.flexnetoperations.com/control/thmo/download?element=6306677) software from Thermo Fisher Scientific. It is only approximately 500 lines long and offers the user the option to export full profile data or the vendor generated centroids (along with Noise and charge state estimation).

Shaduf emphasizes self‐description over compression ratio, (e.g., by avoiding delta‐encoding, employed by the designers of the mz5 format) and as a result the files it produces can be browsed easily using pre‐existing HDF5 viewers, such as the platform‐independent HDFView application, which is distributed by the HDF5 Group (https://www.hdfgroup.org/products/java/hdfview/). Figure 1 shows the ability to inspect individual peak entries along with the ability to observe scan filter data as well as the basepeak chromatogram. Note that the bpc dataset has an attribute named MS:1000628 with a value of “basepeak chromatogram.” In fact, the presence of a red “A” in the dataset icons represents the fact that attributes are present (which, in our case always corresponds to a PSI‐MS CV entry being applied).

## VIII. ACCESSING HDF5 DATA WITH STANDARD AND CUSTOM APIs

**Figure 2:**
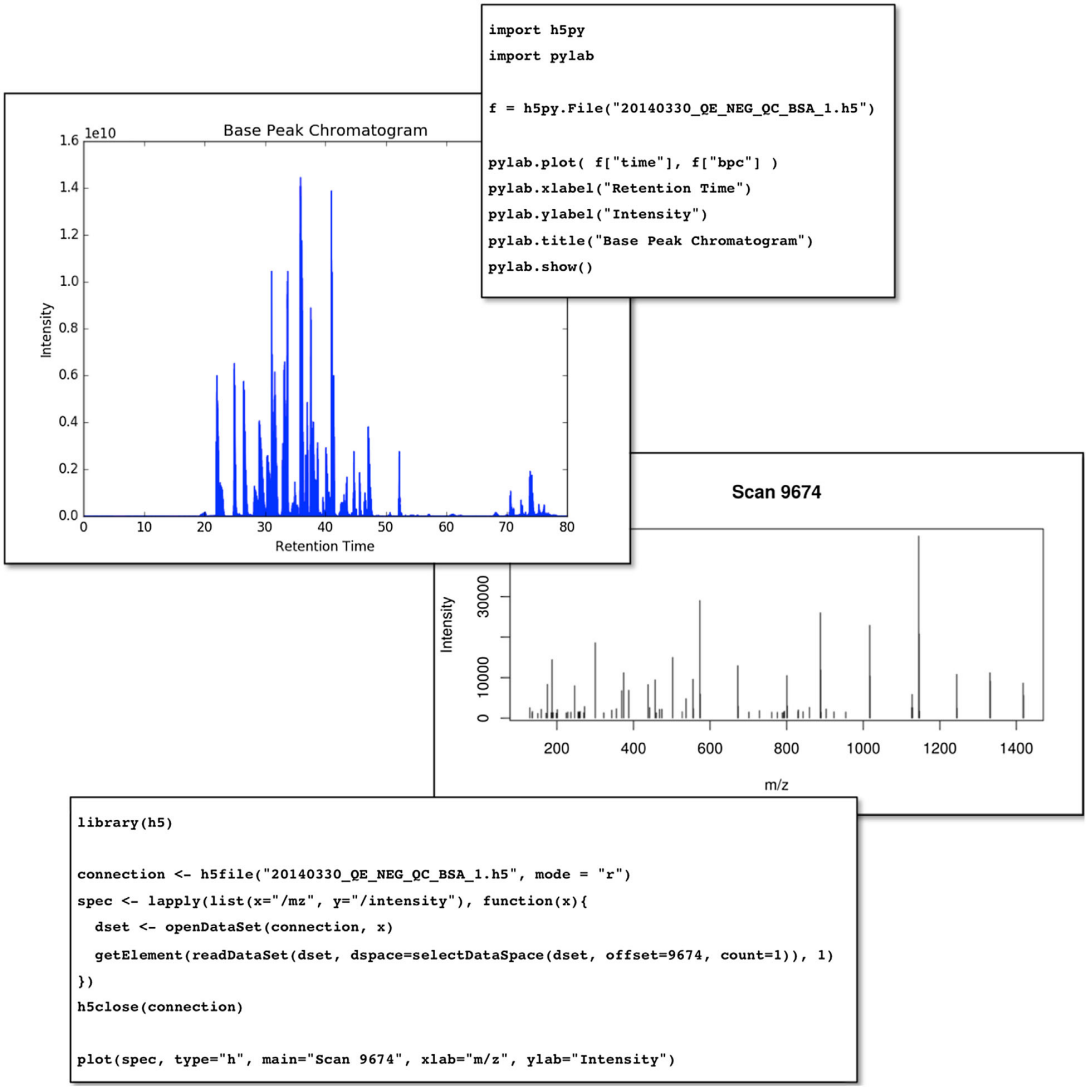
Accessing the same HDF5 file using both the Python and R scripting languages to produce a base peak chromatogram and an individual spectrum, respectively. The spectrum in question is an ms/ms spectrum of a doubly charged precursor ion at 858.92 m/z which corresponds to peptide DALSSVQESQVAQQAR from Bovine Apolipoprotein C‐III.

HDF5 is very well supported across programming languages of interest to the scientific community. The resulting ease of access to HDF5 files is demonstrated for Python (Ramalho, 2015) and R (R Core Team, 2016) with the following snippets, using the standard h5py (Collette, 2014) and h5 (Annau, 2016) Libraries to access and plot the base peak chromatogram and an individual spectrum contained in the file (Fig. 2).

Another example where HDF5 can be used with minimal overhead is in providing remote access to individual scans from large data files using h5serv (https://www.hdfgroup.org/projects/hdfserver/), a RESTful (Richardson & Ruby, 2007) API, and server implementation for remote access to HDF5 files (including the ability to remotely edit the file if necessary).

**Figure 3:**
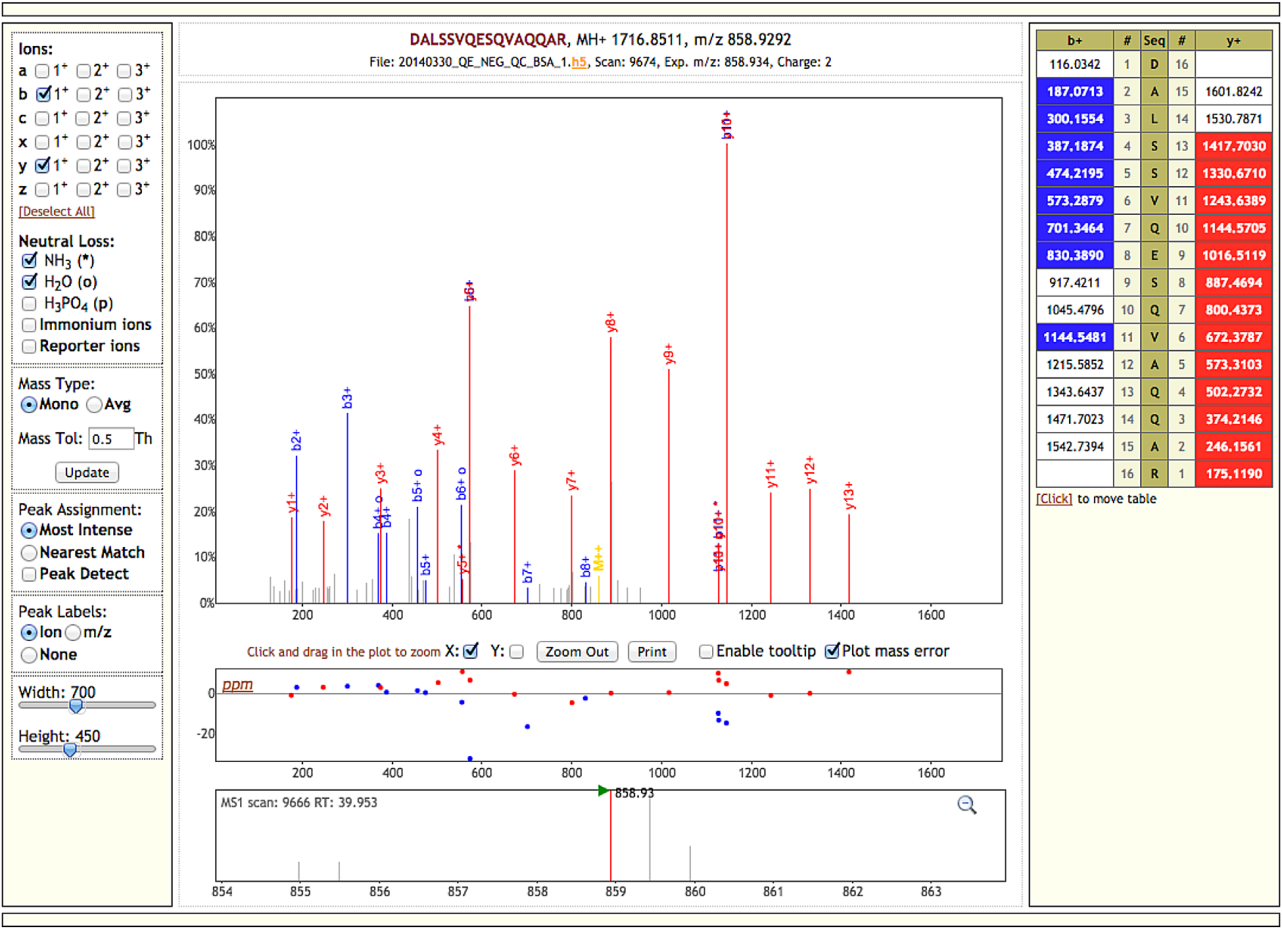
The Lorikeet spectral viewer connected to HDF5‐JSON data provided by h5serv. The ms/ms spectrum being visualized is identical to the one accessed through an R script in the previous figure.

Remote browsing Shaduf generated HDF5 data files using an unmodified h5serv instance and visualizing the data (Fig. 3) with the Lorikeet (Sharma et al., 2012) spectral viewer (http://uwpr.github.io/Lorikeet/) required less than 150 Lines of javascript code to query the h5serv instance and pass the results to Lorikeet. This example demonstrates the benefit of optimizing for self‐description: h5serv is able to provide individual spectra to the client because they are stored explicitly as a set of ragged arrays. Attempting a similar setup with mzML files (using, e.g., a JAX‐RX (Graf, Lewandowski, & Grün, 2010) enabled server) would require additional server‐side code to extract the actual spectral data from the opaque Base64 encoding scheme used to store it.

## IX. CONCLUSION

We have explored a historic trend in mass‐spectrometry data exchange from text‐based representation of individual spectra to increasingly compressed and indexed formats favoring efficiency over human readability. In light of these trends we have highlighted the existing use of HDF5 as a means of preserving universality in a binary context and have demonstrated that a fully self‐describing binary representation of mass spectrometry data can be achieved using plain (“vanilla”) HDF5 as the underlying storage format. A judicious annotation of the resulting datasets with attributes containing PSI‐MS CV accessions yields a candidate interchange format that restores efficient access and storage, scalable up to, and including parallel data handling in HPC data centers. The examples provided, including data access through the standard HDFView file viewer, access from within python and R, as well as remotely through h5serv, all demonstrate that HDF5 constitutes a file format associated with a rich, standard‐compliant computational ecosystem ready to be adopted as the data storage format of choice for the mass spectrometry community. We argue that while use of mzML and related XML‐based formats is possible, a transition to simple (“vanilla”) HDF5 makes data access and sharing effortless, bringing into agreement the requirements of current mass spectrometry data, and storage technology and thus canceling (for a time) the effect of inevitable technical drift.
